# Supplementary material for: Marine Bacterioplankton Community Dynamics and Potentially Pathogenic Bacteria in Seawater around Jeju Island, South Korea, via Metabarcoding
Source: Int J Mol Sci. 2023 Sep 1;24(17):13561. doi: 10.3390/ijms241713561 (PMC10487856; doi:10.3390/ijms241713561)
Supplement: Supplementary file 1 [file ijms-24-13561-s001.zip › ijms-2578030-supplementary.pdf]

Supplementary Table S1. LEfSe analysis for each seawater. ASVs (relative abundance > 0.1% in at least one sample) between September and others (average value of March, May, December).

Taxonomic classification		Months	LDA score (log 10)	p value
Bacteria.Bacteroidetes.Flavobacteriia.Flavobacteriales.Crocinitomicaceae.Fluviicola	2.92	Others	2.90	0.05
Bacteria.Bacteroidetes.Flavobacteriia.Flavobacteriales.Crocinitomicaceae.Fluviicola.Fluviicolahefeinensis	2.92	Others	2.90	0.05
Bacteria.Proteobacteria.Gammaproteobacteria.unclassifiedGammaproteobacteria.unclassifiedGammaproteobacteria.Thiolapillus.Thiolapillusbrandeum	3.20	Others	2.98	0.05
Bacteria.Proteobacteria.Gammaproteobacteria.unclassifiedGammaproteobacteria.unclassifiedGammaproteobacteria.Thiolapillus	3.20	Others	3.00	0.05
Bacteria.Proteobacteria.Gammaproteobacteria.unclassifiedGammaproteobacteria	3.20	Others	3.00	0.05
Bacteria.Proteobacteria.Gammaproteobacteria.unclassifiedGammaproteobacteria.unclassifiedGammaproteobacteria	3.20	Others	3.01	0.05
Bacteria.Proteobacteria.Gammaproteobacteria.Alteromonadales.Colwelliaceae.Colwellia.Colwelliaaestuarii	3.00	Others	3.04	0.04
Bacteria.Proteobacteria.Gammaproteobacteria.Oceanospirillales.Saccharospirillaceae	3.08	Others	3.07	0.05
Bacteria.Proteobacteria.Gammaproteobacteria.Oceanospirillales.Saccharospirillaceae.Reinekea.Reinekeaaestuarii	3.08	Others	3.07	0.05
Bacteria.Proteobacteria.Gammaproteobacteria.Oceanospirillales.Saccharospirillaceae.Reinekea	3.08	Others	3.07	0.05
Bacteria.Proteobacteria.Gammaproteobacteria.Oceanospirillales.Oceanospirillaceae.Neptunomonas.Neptunomonasnaphthovorans	3.31	Others	3.11	0.05
Bacteria.Proteobacteria.Gammaproteobacteria.Vibrionales.Vibrionaceae.Vibrio.Vibriosplendidus	3.28	Others	3.12	0.05
Bacteria.Bacteroidetes.Flavobacteriia.Flavobacteriales.Flavobacteriaceae.Algibacter.Algibacteraestuarii	3.30	Others	3.12	0.04
Bacteria.Proteobacteria.Gammaproteobacteria.Alteromonadales.Alteromonadaceae.Glaciecola.Glaciecolachathamensis	3.15	Others	3.15	0.04
Bacteria.Proteobacteria.Gammaproteobacteria.Alteromonadales.Alteromonadaceae.Glaciecola	3.26	Others	3.16	0.04
Bacteria.Bacteroidetes.Flavobacteriia.Flavobacteriales.Flavobacteriaceae.Polaribacter.Polaribacterlacunae	3.51	Others	3.16	0.05
Bacteria.Proteobacteria.Alphaproteobacteria.Rhodobacterales.Rhodobacteraceae.Amylibacter.Amylibactercionae	2.80	Others	3.16	0.04
Bacteria.Bacteroidetes.Flavobacteriia.Flavobacteriales.Flavobacteriaceae.Polaribacter.Polaribacteratrinae	3.58	Others	3.25	0.05
Bacteria.Proteobacteria.Alphaproteobacteria.Rhodobacterales.Rhodobacteraceae.Amylibacter	3.60	Others	3.27	0.05
Bacteria.Bacteroidetes.Flavobacteriia.Flavobacteriales.Flavobacteriaceae.Algibacter	3.58	Others	3.32	0.04
Bacteria.Proteobacteria.Gammaproteobacteria.Oceanospirillales.Oceanospirillaceae.Oleispira.Oleispiraantarctica	3.62	Others	3.37	0.04
Bacteria.Proteobacteria.Gammaproteobacteria.Oceanospirillales.Oceanospirillaceae.Oleispira	3.62	Others	3.37	0.04
Bacteria.Proteobacteria.Gammaproteobacteria.Alteromonadales.Colwelliaceae.Colwellia	3.69	Others	3.46	0.05
Bacteria.Proteobacteria.Gammaproteobacteria.Alteromonadales.Pseudoalteromonadaceae.Pseudoalteromonas.Pseudoalteromonascarrageenovora	3.98	Others	3.70	0.05
Bacteria.Proteobacteria.Alphaproteobacteria.Rhodobacterales.Rhodobacteraceae.Nereida	4.56	Others	4.16	0.05
Bacteria.Proteobacteria.Alphaproteobacteria.Rhodobacterales.Rhodobacteraceae.Nereida.Nereidaignava	4.56	Others	4.16	0.05
Bacteria.Proteobacteria.Alphaproteobacteria.Rhodobacterales.Rhodobacteraceae.Lentibacter	4.55	Others	4.17	0.05
Bacteria.Proteobacteria.Alphaproteobacteria.Rhodobacterales.Rhodobacteraceae.Lentibacter.Lentibacteralgarum	4.55	Others	4.17	0.05
Bacteria.Bacteroidetes	5.11	Others	4.52	0.05
Bacteria.Bacteroidetes.Flavobacteriia	5.11	Others	4.56	0.05
Bacteria.Bacteroidetes.Flavobacteriia.Flavobacteriales.Flavobacteriaceae	5.09	Others	4.56	0.05
Bacteria.Bacteroidetes.Flavobacteriia.Flavobacteriales.Flavobacteriaceae.Aurantivirga.Aurantivirgaprofunda	4.89	Others	4.57	0.05
Bacteria.Bacteroidetes.Flavobacteriia.Flavobacteriales.Flavobacteriaceae.Aurantivirga	4.89	Others	4.57	0.05
Bacteria.Bacteroidetes.Flavobacteriia.Flavobacteriales	5.11	Others	4.57	0.05
Bacteria.Proteobacteria.Gammaproteobacteria.Alteromonadales	5.34	Others	4.80	0.05
Bacteria.Proteobacteria.Gammaproteobacteria.Alteromonadales.Alteromonadaceae.Aestuariibacter	5.25	Others	4.91	0.05

Bacteria.Proteobacteria.Gammaproteobacteria.Alteromonadales.Alteromonadaceae.Aestuariibacter.Aestuariibacterhalophilus	5.25	Others	4.91	0.05
Bacteria.Proteobacteria.Gammaproteobacteria.Alteromonadales.Alteromonadaceae	5.29	Others	4.93	0.05
Bacteria.Proteobacteria.Alphaproteobacteria.Rhodobacterales.Rhodobacteraceae.Ruegeria.Ruegeriaconchae	2.78	September	2.75	0.01
Bacteria.Proteobacteria.Alphaproteobacteria.Rhodobacterales.Rhodobacteraceae.Roseicyclus.Roseicyclusmahoneyensis	2.85	September	2.75	0.01
Bacteria.Proteobacteria.Alphaproteobacteria.Rhodobacterales.Rhodobacteraceae.Roseicyclus	2.85	September	2.76	0.01
Bacteria.Proteobacteria.Gammaproteobacteria.Vibrionales.Vibrionaceae.Vibrio.Vibrioowensii	2.82	September	2.76	0.01
Bacteria.Bacteroidetes.Flavobacteriia.Flavobacteriales.Flavobacteriaceae.Mesoflavibacter.Mesoflavibactersabulilitoris	2.89	September	2.77	0.01
Bacteria.Proteobacteria.Gammaproteobacteria.Alteromonadales.Pseudoalteromonadaceae.Pseudoalteromonas.Pseudoalteromonasrubra	2.89	September	2.81	0.02
Bacteria.Proteobacteria.Alphaproteobacteria.Rhodobacterales.Rhodobacteraceae.Roseovarius.Roseovariuscrassostreae	2.85	September	2.82	0.01
Bacteria.Proteobacteria.Gammaproteobacteria.Alteromonadales.Pseudoalteromonadaceae.Pseudoalteromonas.Pseudoalteromonasprofundi	2.92	September	2.82	0.01
Bacteria.Proteobacteria.Gammaproteobacteria.Alteromonadales.Pseudoalteromonadaceae.Pseudoalteromonas.Pseudoalteromonasspongiae	2.82	September	2.84	0.03
Bacteria.Proteobacteria.Alphaproteobacteria.Sphingomonadales.Erythrobacteraceae.Erythrobacter.Erythrobactergaetbuli	2.92	September	2.85	0.01
Bacteria.Bacteroidetes.Flavobacteriia.Flavobacteriales.Flavobacteriaceae.Mesonia	3.04	September	2.86	0.01
Bacteria.Bacteroidetes.Flavobacteriia.Flavobacteriales.Flavobacteriaceae.Mesonia.Mesoniamobilis	3.04	September	2.87	0.01
Bacteria.Bacteroidetes.Flavobacteriia.Flavobacteriales.Flavobacteriaceae.Muricauda.Muricaudabeolgyonensis	3.30	September	2.89	0.03
Bacteria.Proteobacteria.Gammaproteobacteria.Vibrionales.Vibrionaceae.Vibrio.Vibrioponticus	2.92	September	2.90	0.02
Bacteria.Proteobacteria.Gammaproteobacteria.Vibrionales.Vibrionaceae.Vibrio.Vibrioplantisponsor	2.89	September	2.91	0.03
Bacteria.Proteobacteria.Gammaproteobacteria.Vibrionales.Vibrionaceae.Vibrio.Vibriocampbellii	3.14	September	2.92	0.03
Bacteria.Proteobacteria.Alphaproteobacteria.Rhodobacterales.Rhodobacteraceae.Citreicella	3.14	September	2.93	0.01
Bacteria.Proteobacteria.Alphaproteobacteria.Sphingomonadales.Erythrobacteraceae.Erythrobacter.Erythrobacterlutimaris	3.08	September	2.93	0.01
Bacteria.Proteobacteria.Gammaproteobacteria.Pseudomonadales.Moraxellaceae.Psychrobacter.Psychrobactermarincola	3.06	September	2.93	0.01
Bacteria.Proteobacteria.Alphaproteobacteria.Rhodobacterales.Rhodobacteraceae.Paracoccus.Paracoccus_uc	3.17	September	2.93	0.03
Bacteria.Bacteroidetes.Flavobacteriia.Flavobacteriales.Flavobacteriaceae.Muricauda	3.30	September	2.93	0.03
Bacteria.Bacteroidetes.Flavobacteriia.Flavobacteriales.Flavobacteriaceae.Bizionia.Bizioniahallyeonensis	3.08	September	2.94	0.01
Bacteria.Proteobacteria.Alphaproteobacteria.Rhodobacterales.Rhodobacteraceae.Citreicella.Citreicellathiooxidans	3.14	September	2.94	0.01
Bacteria.Proteobacteria.Alphaproteobacteria.Rhizobiales.Hyphomicrobiaceae.Devosia	3.41	September	2.95	0.05
Bacteria.Proteobacteria.Gammaproteobacteria.Alteromonadales.Colwelliaceae.Thalassotalea.Thalassotalealoyana	3.28	September	2.96	0.04
Bacteria.Proteobacteria.Alphaproteobacteria.Rhizobiales.Hyphomicrobiaceae.Devosia.Devosialimi	3.41	September	2.97	0.05
Bacteria.Proteobacteria.Alphaproteobacteria.Rhodobacterales.Rhodobacteraceae.Pelagibaca.Pelagibacabermudensis	3.17	September	2.99	0.01
Bacteria.Bacteroidetes.Flavobacteriia.Flavobacteriales.Flavobacteriaceae.Dokdonia.Dokdonialutea	3.12	September	2.99	0.03
Bacteria.Proteobacteria.Alphaproteobacteria.Kiloniellales.Kiloniellaceae.Kiloniella.Kiloniellalitopenaei	3.30	September	2.99	0.05
Bacteria.Proteobacteria.Alphaproteobacteria.Rhodobacterales.Rhodobacteraceae.Rhodobacter.Rhodobactercapsulatus	3.26	September	3.00	0.01
Bacteria.Bacteroidetes.Flavobacteriia.Flavobacteriales.Flavobacteriaceae.Dokdonia	3.12	September	3.00	0.03
Bacteria.Proteobacteria.Alphaproteobacteria.Kiloniellales.Kiloniellaceae	3.30	September	3.01	0.05
Bacteria.Proteobacteria.Alphaproteobacteria.Kiloniellales.Kiloniellaceae.Kiloniella	3.30	September	3.01	0.05
Bacteria.Proteobacteria.Alphaproteobacteria.Kiloniellales	3.30	September	3.01	0.05
Bacteria.Proteobacteria.Gammaproteobacteria.Vibrionales.Vibrionaceae.Vibrio.Vibrioxuii	3.27	September	3.02	0.01
Bacteria.Proteobacteria.Gammaproteobacteria.Vibrionales.Vibrionaceae.Vibrio.Vibriofortis	3.30	September	3.04	0.02
Bacteria.Proteobacteria.Alphaproteobacteria.Rhodobacterales.Rhodobacteraceae.Sulfitobacter.Sulfitobacterdubius	3.28	September	3.04	0.04
Bacteria.Proteobacteria.Gammaproteobacteria.Vibrionales.Vibrionaceae.Vibrio.Vibriocommunis	3.06	September	3.05	0.01
Bacteria.Proteobacteria.Alphaproteobacteria.Rhodobacterales.Rhodobacteraceae.Oceanicola.Oceanicolalitoreus	3.16	September	3.05	0.01
Bacteria.Bacteroidetes.Flavobacteriia.Flavobacteriales.Flavobacteriaceae.Hyunsoonleella	3.06	September	3.06	0.04
Bacteria.Proteobacteria.Alphaproteobacteria.Rhizobiales.Cohaesibacteraceae.Cohaesibacter.Cohaesibactermarisflavi	3.37	September	3.06	0.01

Bacteria.Proteobacteria.Gammaproteobacteria.Alteromonadales.Colwelliaceae.Thalassotalea.Thalassotaleafusca	3.34	September	3.07	0.04
Bacteria.Proteobacteria.Alphaproteobacteria.Rhodobacterales.Rhodobacteraceae.Oceanicola	3.38	September	3.08	0.04
Bacteria.Proteobacteria.Alphaproteobacteria.Rhodobacterales.Rhodobacteraceae.Nioella.Nioellaaestuarii	3.10	September	3.08	0.02
Bacteria.Bacteroidetes.Flavobacteriia.Flavobacteriales.Flavobacteriaceae.Gilvibacter	3.35	September	3.08	0.01
Bacteria.Proteobacteria.Alphaproteobacteria.Sphingomonadales.Erythrobacteraceae.Altererythrobacter.Altererythrobacterishigakiens is	3.34	September	3.09	0.01
Bacteria.Bacteroidetes.Flavobacteriia.Flavobacteriales.Flavobacteriaceae.Salinimicrobium.Salinimicrobiumsoli	3.33	September	3.09	0.01
Bacteria.Proteobacteria.Gammaproteobacteria.Alteromonadales.Pseudoalteromonadaceae.Pseudoalteromonas.Pseudoalteromonaspry dzensis	3.30	September	3.09	0.02
Bacteria.Bacteroidetes.Flavobacteriia.Flavobacteriales.Flavobacteriaceae.Hyunsoonleella.Hyunsoonleellaudonensis	3.06	September	3.09	0.04
Bacteria.Bacteroidetes.Flavobacteriia.Flavobacteriales.Flavobacteriaceae.Salinimicrobium	3.33	September	3.10	0.01
Bacteria.Bacteroidetes.Flavobacteriia.Flavobacteriales.Flavobacteriaceae.Gilvibacter.Gilvibactersediminis	3.35	September	3.10	0.01
Bacteria.Proteobacteria.Alphaproteobacteria.Rhodobacterales.Rhodobacteraceae.Nioella	3.10	September	3.11	0.02
Bacteria.Proteobacteria.Alphaproteobacteria.Rhizobiales.Hyphomicrobiaceae	3.66	September	3.11	0.05
Bacteria.Proteobacteria.Alphaproteobacteria.Sphingomonadales.Erythrobacteraceae.Altererythrobacter	3.34	September	3.12	0.01
Bacteria.Proteobacteria.Gammaproteobacteria.Vibrionales.Vibrionaceae.Vibrio.Vibriopelagius	3.42	September	3.12	0.01
Bacteria.Bacteroidetes.Flavobacteriia.Flavobacteriales.Crocinitomicaceae.Salinirepens.Salinirepensamamiensis	3.40	September	3.13	0.05
Bacteria.Bacteroidetes.Flavobacteriia.Flavobacteriales.Flavobacteriaceae.Mesoflavibacter.Mesoflavibacterzeaxanthinifaciens	3.45	September	3.14	0.01
Bacteria.Bacteroidetes.Flavobacteriia.Flavobacteriales.Flavobacteriaceae.Pseudotenacibaculum	3.33	September	3.14	0.01
Bacteria.Bacteroidetes.Flavobacteriia.Flavobacteriales.Crocinitomicaceae.Salinirepens	3.40	September	3.14	0.05
Bacteria.Bacteroidetes.Flavobacteriia.Flavobacteriales.Flavobacteriaceae.Pseudotenacibaculum.Pseudotenacibaculumhaliotis	3.33	September	3.14	0.01
Bacteria.Proteobacteria.Alphaproteobacteria.Rhodobacterales.Rhodobacteraceae.Celeribacter.Celeribactermanganoxidans	3.00	September	3.15	0.02
Bacteria.Proteobacteria.Alphaproteobacteria.Rhodobacterales.Rhodobacteraceae.Donghicola.Donghicolatyrosinivorans	3.47	September	3.17	0.02
Bacteria.Proteobacteria.Alphaproteobacteria.Sphingomonadales.Erythrobacteraceae.Erythrobacter.Erythrobactercitreus	3.39	September	3.17	0.01
Bacteria.Proteobacteria.Alphaproteobacteria.Rhizobiales.Cohaesibacteraceae.Cohaesibacter	3.53	September	3.18	0.02
Bacteria.Proteobacteria.Alphaproteobacteria.Rhodospirillales.Rhodospirillaceae.Thalassospira	3.56	September	3.19	0.02
Bacteria.Bacteroidetes.Flavobacteriia.Flavobacteriales.Flavobacteriaceae.Croceitalea.Croceitaleaeckloniae	3.14	September	3.19	0.02
Bacteria.Bacteroidetes.Flavobacteriia.Flavobacteriales.Flavobacteriaceae.Croceitalea	3.14	September	3.20	0.02
Bacteria.Proteobacteria.Alphaproteobacteria.Rhizobiales.Cohaesibacteraceae	3.53	September	3.21	0.02
Bacteria.Proteobacteria.Alphaproteobacteria.Rhodospirillales.Rhodospirillaceae.Thalassospira.Thalassospiratepidiphila	3.56	September	3.22	0.02
Bacteria.Proteobacteria.Gammaproteobacteria.Alteromonadales.Pseudoalteromonadaceae.Pseudoalteromonas.Pseudoalteromonaslut eoviolacea	3.54	September	3.22	0.05
Bacteria.Proteobacteria.Alphaproteobacteria.Rhodobacterales.Rhodobacteraceae.Marivita.Marivitabyunsanensis	3.26	September	3.22	0.04
Bacteria.Proteobacteria.Alphaproteobacteria.Rhodobacterales.Rhodobacteraceae.Pelagibaca.Pelagibacaabyssi	3.47	September	3.24	0.02
Bacteria.Bacteroidetes.Flavobacteriia.Flavobacteriales.Flavobacteriaceae.Mesoflavibacter	3.55	September	3.25	0.01
Bacteria.Proteobacteria.Gammaproteobacteria.Vibrionales.Vibrionaceae.Vibrio.Vibriomaritimus	3.56	September	3.25	0.02
Bacteria.Cyanobacteria.Cyanophyceae.Synechococcales.Prochloraceae	3.54	September	3.27	0.02
Bacteria.Cyanobacteria.Cyanophyceae.Synechococcales.Prochloraceae.Prochlorococcus.Prochlorococcusmarinus	3.54	September	3.27	0.02
Bacteria.Proteobacteria.Alphaproteobacteria.Rhodospirillales.Rhodospirillaceae	3.76	September	3.29	0.05
Bacteria.Proteobacteria.Gammaproteobacteria.Cellvibrionales.Halieaceae.Luminiphilus	3.57	September	3.30	0.05
Bacteria.Cyanobacteria.Cyanophyceae.Synechococcales.Prochloraceae.Prochlorococcus	3.54	September	3.30	0.02
Bacteria.Proteobacteria.Alphaproteobacteria.Rhodospirillales	3.76	September	3.31	0.05
Bacteria.Proteobacteria.Alphaproteobacteria.Rhodobacterales.Rhodobacteraceae.Rhodobacter	3.54	September	3.31	0.01
Bacteria.Bacteroidetes.Flavobacteriia.Flavobacteriales.Flavobacteriaceae.Formosa	3.69	September	3.32	0.05
Bacteria.Proteobacteria.Alphaproteobacteria.Rhodobacterales.Rhodobacteraceae.Ahrensia.Ahrensiakielensis	3.62	September	3.32	0.04
Bacteria.Proteobacteria.Alphaproteobacteria.Rhodobacterales.Rhodobacteraceae.Ahrensia	3.62	September	3.34	0.04

Bacteria.Bacteroidetes.Saprospiria.Saprospirales.Haliscomenobacteraceae.Phaeodactylibacter.Phaeodactylibacterxiamenensis	3.58	September	3.34	0.02
Bacteria.Bacteroidetes.Saprospiria.Saprospirales.Haliscomenobacteraceae.Phaeodactylibacter	3.58	September	3.35	0.02
Bacteria.Bacteroidetes.Flavobacteriia.Flavobacteriales.Flavobacteriaceae.Formosa.Formosaalgae	3.69	September	3.36	0.05
Bacteria.Proteobacteria.Gammaproteobacteria.Cellvibrionales.Halieaceae.Luminiphilus.Luminiphilussyltensis	3.57	September	3.36	0.05
Bacteria.Proteobacteria.Alphaproteobacteria.Rhodobacterales.Rhodobacteraceae.Pelagibaca	3.65	September	3.36	0.02
Bacteria.Proteobacteria.Gammaproteobacteria.Pseudomonadales.Moraxellaceae.Psychrobacter.Psychrobacterceler	3.63	September	3.36	0.03
Bacteria.Proteobacteria.Gammaproteobacteria.Vibrionales.Vibrionaceae.Vibrio.Vibriorumoiensis	3.76	September	3.38	0.05
Bacteria.Proteobacteria.Alphaproteobacteria.Rhodobacterales.Rhodobacteraceae.Shimia.Shimiaisoporae	3.99	September	3.38	0.05
Bacteria.Proteobacteria.Alphaproteobacteria.Rhodobacterales.Rhodobacteraceae.Yangia	3.65	September	3.39	0.01
Bacteria.Bacteroidetes.Saprospiria	3.58	September	3.39	0.02
Bacteria.Proteobacteria.Alphaproteobacteria.Rhodobacterales.Rhodobacteraceae.Shimia	3.99	September	3.39	0.05
Bacteria.Proteobacteria.Alphaproteobacteria.Rhodobacterales.Rhodobacteraceae.Yangia.Yangiapacifica	3.65	September	3.39	0.01
Bacteria.Proteobacteria.Gammaproteobacteria.Alteromonadales.Pseudoalteromonadaceae.Pseudoalteromonas.Pseudoalteromonasarctica	3.75	September	3.40	0.04
Bacteria.Proteobacteria.Gammaproteobacteria.Alteromonadales.Colwelliaceae.Thalassotalea	3.62	September	3.44	0.05
Bacteria.Proteobacteria.Alphaproteobacteria.Rhodobacterales.Rhodobacteraceae.Celeribacter.Celeribacterbaekdonensis	3.76	September	3.46	0.04
Bacteria.Proteobacteria.Gammaproteobacteria.Pseudomonadales.Moraxellaceae	3.74	September	3.48	0.03
Bacteria.Proteobacteria.Alphaproteobacteria.Sphingomonadales.Erythrobacteraceae.Erythrobacter.Erythrobacterseohaensis	3.78	September	3.51	0.02
Bacteria.Proteobacteria.Alphaproteobacteria.Rhodobacterales.Rhodobacteraceae.Celeribacter	3.88	September	3.51	0.04
Bacteria.Proteobacteria.Alphaproteobacteria.Rhodobacterales.Rhodobacteraceae.Aestuariicoccus	3.81	September	3.52	0.02
Bacteria.Proteobacteria.Alphaproteobacteria.Rhodobacterales.Rhodobacteraceae.Leisingera.Leisingeracaerulea	4.07	September	3.53	0.05
Bacteria.Proteobacteria.Alphaproteobacteria.Rhodobacterales.Rhodobacteraceae.Nautella	3.90	September	3.56	0.05
Bacteria.Proteobacteria.Alphaproteobacteria.Rhodobacterales.Rhodobacteraceae.Nautella.Nautellaitalica	3.90	September	3.57	0.05
Bacteria.Proteobacteria.Alphaproteobacteria.Rhodobacterales.Rhodobacteraceae.Aestuariicoccus.Aestuariicoccusmarinus	3.81	September	3.61	0.02
Bacteria.Proteobacteria.Gammaproteobacteria.Vibrionales.Vibrionaceae.Vibrio.Vibriotasmaniensis	3.94	September	3.62	0.05
Bacteria.Proteobacteria.Gammaproteobacteria.Vibrionales.Vibrionaceae.Vibrio.Vibriochagasii	3.98	September	3.63	0.05
Bacteria.Proteobacteria.Gammaproteobacteria.Alteromonadales.Pseudoalteromonadaceae.Pseudoalteromonas.Pseudoalteromonasnigrifaciens	4.02	September	3.66	0.05
Bacteria.Proteobacteria.Gammaproteobacteria.Pseudomonadales.Moraxellaceae.Psychrobacter	3.74	September	3.67	0.03
Bacteria.Proteobacteria.Alphaproteobacteria.Rhodobacterales.Rhodobacteraceae.Donghicola.Donghicolaeburneus	3.97	September	3.67	0.02
Bacteria.Proteobacteria.Gammaproteobacteria.Pseudomonadales	3.74	September	3.67	0.03
Bacteria.Bacteroidetes.Saprospiria.Saprospirales.Haliscomenobacteraceae	3.58	September	3.70	0.02
Bacteria.Bacteroidetes.Saprospiria.Saprospirales	3.58	September	3.71	0.02
Bacteria.Proteobacteria.Gammaproteobacteria.Alteromonadales.Pseudoalteromonadaceae.Pseudoalteromonas.Pseudoalteromonasagarivorans	4.11	September	3.74	0.05
Bacteria.Proteobacteria.Alphaproteobacteria.Sphingomonadales.Erythrobacteraceae.Erythrobacter.Erythrobacterflavus	4.05	September	3.75	0.02
Bacteria.Proteobacteria.Alphaproteobacteria.Rhodobacterales.Rhodobacteraceae.Donghicola	4.09	September	3.76	0.03
Bacteria.Proteobacteria.Gammaproteobacteria.Alteromonadales.Pseudoalteromonadaceae.Pseudoalteromonas.Pseudoalteromonasarabiensis	4.06	September	3.79	0.02
Bacteria.Proteobacteria.Gammaproteobacteria.Vibrionales.Vibrionaceae.Vibrio.Vibrioshilonii	4.08	September	3.79	0.02
Bacteria.Proteobacteria.Alphaproteobacteria.Rhodobacterales.Rhodobacteraceae.Marivita.Marivitageojedonensis	4.22	September	3.81	0.05
Bacteria.Proteobacteria.Alphaproteobacteria.Rhodobacterales.Rhodobacteraceae.Marivita	4.26	September	3.87	0.05
Bacteria.Proteobacteria.Alphaproteobacteria.Rhodobacterales.Rhodobacteraceae.Phaeobacter.Phaeobacteritalicus	4.25	September	3.90	0.05
Bacteria.Proteobacteria.Gammaproteobacteria.Alteromonadales.Pseudoalteromonadaceae.Pseudoalteromonas.Pseudoalteromonastetraodonis	4.25	September	3.93	0.05
Bacteria.Proteobacteria.Gammaproteobacteria.Vibrionales.Vibrionaceae.Vibrio.Vibriokanaloae	4.26	September	3.94	0.04

Bacteria.Cyanobacteria.Cyanophyceae.Synechococcales.Synechococcaceae.Synechococcus.Synechococcusrubescens	4.55	September	4.05	0.05
Bacteria.Proteobacteria.Alphaproteobacteria.Sphingomonadales.Erythrobacteraceae.Erythrobacter	4.33	September	4.06	0.02
Bacteria.Cyanobacteria.Cyanophyceae.Synechococcales.Synechococcaceae	4.55	September	4.12	0.05
Bacteria.Cyanobacteria.Cyanophyceae.Synechococcales.Synechococcaceae.Synechococcus	4.55	September	4.14	0.05
Bacteria.Cyanobacteria.Cyanophyceae	4.59	September	4.16	0.05
Bacteria.Cyanobacteria	4.59	September	4.16	0.05
Bacteria.Proteobacteria.Alphaproteobacteria.Rhodobacterales.Rhodobacteraceae.Marivivens.Marivivensdonghaensis	4.49	September	4.18	0.01
Bacteria.Cyanobacteria.Cyanophyceae.Synechococcales	4.59	September	4.20	0.05
Bacteria.Proteobacteria.Alphaproteobacteria.Rhodobacterales.Rhodobacteraceae.Marivivens	4.49	September	4.21	0.01
Bacteria.Proteobacteria.Gammaproteobacteria.Alteromonadales.Pseudoalteromonadaceae	4.84	September	4.36	0.05
Bacteria.Proteobacteria.Gammaproteobacteria.Alteromonadales.Pseudoalteromonadaceae.Pseudoalteromonas	4.83	September	4.37	0.05
Bacteria.Proteobacteria.Alphaproteobacteria.Sphingomonadales.Erythrobacteraceae.Citromicrobium.Citromicrobiumbathyomarinum	4.83	September	4.52	0.01
Bacteria.Proteobacteria.Alphaproteobacteria.Sphingomonadales.Erythrobacteraceae.Citromicrobium	4.83	September	4.54	0.01
Bacteria.Proteobacteria.Alphaproteobacteria.Sphingomonadales	4.97	September	4.68	0.05
Bacteria.Proteobacteria.Alphaproteobacteria.Sphingomonadales.Erythrobacteraceae	4.96	September	4.68	0.02

Supplementary Table S2. Summary of potential pathogenic bacteria and their hosts.

Class	Species	Host	Reference
Betaproteobacteria	*Comamonas testosteroni*		[1]
Epsilonproteobacteria	*Arcobacter cryaerophilus*	human, terrestrial animal (poultry, porcine, bovine, sheep feces), clams	[2-4]
Flavobacteriia	*Formosa algae*	macroalgae	[5]
Flavobacteriia	*Tenacibaculum discolor*		[6]
Flavobacteriia	*Tenacibaculum soleae*	fish (*Trachurus trachurus*)	[7]
Gammaproteobacteria	*Acinetobacter johnsonii*	human; fish (rainbow trout)	[8, 9]
Gammaproteobacteria	*Acinetobacter venetianus*	whiteleg shrimp	[10]
Gammaproteobacteria	*Aeromonas hydrophila*		[11]
Gammaproteobacteria	*Coxiella burnetii*	human	[12]
Gammaproteobacteria	*Moritella viscosa*	fish (salmonid)	[13]
Gammaproteobacteria	*Photobacterium damselae*		[14]
Gammaproteobacteria	*Photobacterium iliopiscarium*	fish	[15]
Gammaproteobacteria	*Photobacterium rosenbergii*	sponges	[15]
Gammaproteobacteria	*Photobacterium swingsii*	fish (torafugu), octopus (*Octopus vulgaris*)	[16]
Gammaproteobacteria	*Pseudoalteromonas arctica*	red alga (*Agarophyton vermiculophyllum*)	[17]
Gammaproteobacteria	*Pseudoalteromonas elyakovii*	macroalga	[5]
Gammaproteobacteria	*Pseudoalteromonas espejiana*	jellyfish (*Aurelia aurita*)	[18]
Gammaproteobacteria	*Pseudoalteromonas marina*	red algae (*Pyropia yezoensis*)	[19]
Gammaproteobacteria	*Pseudoalteromonas nigrifaciens*	sea cucumber (*Apostichopus japonicus*)	[20]
Gammaproteobacteria	*Pseudoalteromonas tetraodonis*	fish	[21-23]
Gammaproteobacteria	*Shewanella baltica*	fish (*Danio rerio*)	[24]
Gammaproteobacteria	*Vibrio aestuarianus*	oyster	[25]
Gammaproteobacteria	*Vibrio alfacsensis*	fish	[26]
Gammaproteobacteria	*Vibrio brasiliensis*	shrimp (*Litopenaeus vannamei*)	[27, 28]
Gammaproteobacteria	*Vibrio campbellii*	shrimp (*Litopenaeus vannamei*)	[29]
Gammaproteobacteria	*Vibrio chagasii*	oyster (*Crassostrea gigas*), fish (salmonids)	[30, 31]
Gammaproteobacteria	*Vibrio crassostreae*	oyster	[32]
Gammaproteobacteria	*Vibrio diabolicus*	clam (*Gomphina aequilatera*)	[33]
Gammaproteobacteria	*Vibrio fluvialis*	Human	[34]
Gammaproteobacteria	*Vibrio fortis*	fish (seahorses)	[35]
Gammaproteobacteria	*Vibrio gigantis*	human, oyster (*Crassostrea gigas*)	[36]
Gammaproteobacteria	*Vibrio harveyi*	fish (*Seriola lalandi* and *S. dumerili*)	[37]
Gammaproteobacteria	*Vibrio jasicida*	moth (*Galleria mellonella*)	[38]
Gammaproteobacteria	*Vibrio kanaloae*	lobster (*Jasus verreauxi*)	[39]
Gammaproteobacteria	*Vibrio lentus*	clam (*Scapharca broughtonii*), fish (*Dicentrarchus labrax*), octopus (*Octopus vulgaris*)	[40, 41]
Gammaproteobacteria	*Vibrio mediterranei*	mussel (*Pinna nobilis*), manila clams, brine shrimp	[42, 43]
Gammaproteobacteria	*Vibrio nereis*	shrimp	[44]
Gammaproteobacteria	*Vibrio ordalii*	fish (salmonids)	[45]
Gammaproteobacteria	*Vibrio owensii*	coral	[46]
Gammaproteobacteria	*Vibrio pelagius*	fish (*Scophthalmus maximus*)	[47]
Gammaproteobacteria	*Vibrio pomeroyi*	clams	[48]

Gammaproteobacteria	*Vibrio proteolyticus*	shrimp (*Artemia* spp.), human	[49, 50]
Gammaproteobacteria	*Vibrio sagamiensis*	fish (turbot, sole)	[51]
Gammaproteobacteria	*Vibrio scophthalmi*	fish (*Paralichthys olivaceus*)	[52]
Gammaproteobacteria	*Vibrio splendidus*	oyster (*Crassostrea gigas*)	[53, 54]
Gammaproteobacteria	*Vibrio tapetis*	clams	[48]
Gammaproteobacteria	*Vibrio tasmaniensis*	oyster	[55]
Gammaproteobacteria	*Vibrio vulnificus*	human	[56]
Gammaproteobacteria	*Vibrio xuii*	fish (turbot, sole)	[14]

Selected References

1. Yang, M.; Wang, Q.; Chen, J.; Wu, H., The occurrence of potential pathogenic bacteria on international ships' ballast water at Yangshan Port, Shanghai, China. *Marine Pollution Bulletin* **2022,** 184, 114190.
2. Houf, K.; Stephan, R., Isolation and characterization of the emerging foodborn pathogen Arcobacter from human stool. *Journal of Microbiological Methods* **2007,** 68, (2), 408-413.
3. Johnson, L. G.; Murano, E. A., Arcobacter isolates from various sources. *Journal of food protection* **2002,** 65, (11), 1789-1795.
4. Levican, A.; Alkeskas, A.; Günter, C.; Forsythe, S. J.; Figueras, M. J., Adherence to and invasion of human intestinal cells by Arcobacter species and their virulence genotypes. *Applied and environmental microbiology* **2013,** 79, (16), 4951-4957.
5. Goecke, F.; Labes, A.; Wiese, J.; Imhoff, J. F., Phylogenetic analysis and antibiotic activity of bacteria isolated from the surface of two co-occurring macroalgae from the Baltic Sea. *European journal of phycology* **2013,** 48, (1), 47-60.
6. Pineiro-Vidal, M.; Riaza, A.; Santos, Y., Tenacibaculum discolor sp. nov. and Tenacibaculum gallaicum sp. nov., isolated from sole (Solea senegalensis) and turbot (Psetta maxima) culture systems. *International journal of systematic and evolutionary microbiology* **2008,** 58, (1), 21-25.
7. Fernández-Álvarez, C.; Santos, Y., Phenotypic and Molecular Characterization of Lacinutrix venerupis Isolated from Atlantic Horse Mackerel Trachurus trachurus. *Journal of Aquatic Animal Health* **2019,** 31, (4), 320-327.
8. Kozińska, A.; Paździor, E.; Pękala, A.; Niemczuk, W., Acinetobacter johnsonii and Acinetobacter lwoffii-the emerging fish pathogens. *Journal of Veterinary Research* **2014,** 58, (2), 193-199.
9. Seifert, H.; Strate, A.; Schulze, A.; Pulverer, G., Vascular Catheter—Related Bloodstream Infection Due to Acinetobacter johnsonii (Formerly Acinetobacter calcoaceticus var. lwoffii): Report of 13 Cases. *Clinical Infectious Diseases* **1993,** 17, (4), 632-636.
10. Manan, H.; Rosland, N. A.; Mat Deris, Z.; Che Hashim, N. F.; Kasan, N. A.; Ikhwanuddin, M.; Suloma, A.; Fauzan, F., 16S rRNA sequences of Exiguobacterium spp. bacteria dominant in a biofloc pond cultured with whiteleg shrimp, Penaeus vannamei. *Aquaculture Research* **2022,** 53, (5), 2029-2041.
11. Jalali, S.; Kohli, S.; Latka, C.; Bhatia, S.; Vellarikal, S. K.; Sivasubbu, S.; Scaria, V.; Ramachandran, S., Screening currency notes for microbial pathogens and antibiotic resistance genes using a shotgun metagenomic approach. *PLoS One* **2015,** 10, (6), e0128711.
12. Kazar, J., Coxiella burnetii infection. *Annals of the New York Academy of Sciences* **2005,** 1063, (1), 105-114.
13. Løvoll, M.; Wiik-Nielsen, C.; Tunsjø, H. S.; Colquhoun, D.; Lunder, T.; Sørum, H.; Grove, S., Atlantic salmon bath challenged with Moritella viscosa–pathogen invasion and host response. *Fish & shellfish immunology* **2009,** 26, (6), 877-884.
14. Martins, P.; Cleary, D. F.; Pires, A. C.; Rodrigues, A. M.; Quintino, V.; Calado, R.; Gomes, N. C., Molecular analysis of bacterial communities and detection of potential pathogens in a recirculating aquaculture system for Scophthalmus maximus and Solea senegalensis. *PloS one* **2013,** 8, (11), e80847.
15. Urbanczyk, H.; Ogura, Y.; Hendry, T. A.; Gould, A. L.; Kiwaki, N.; Atkinson, J. T.; Hayashi, T.; Dunlap, P. V., Genome sequence of Photobacterium mandapamensis strain svers. 1.1, the bioluminescent symbiont of the cardinal fish Siphamia versicolor. In Am Soc Microbiol: 2011.
16. Fichi, G.; Cardeti, G.; Perrucci, S.; Vanni, A.; Cersini, A.; Lenzi, C.; De Wolf, T.; Fronte, B.; Guarducci, M.; Susini, F., Skin lesion-associated pathogens from Octopus vulgaris: first detection of Photobacterium swingsii, Lactococcus garvieae and betanodavirus. *Diseases of Aquatic Organisms* **2015,** 115, (2), 147-156.
17. Li, J.; Weinberger, F.; Saha, M.; Majzoub, M. E.; Egan, S., Cross-host protection of marine bacteria against macroalgal disease. *Microbial ecology* **2022,** 84, (4), 1288-1293.
18. Weiland-Bräuer, N.; Pinnow, N.; Langfeldt, D.; Roik, A.; Güllert, S.; Chibani, C. M.; Reusch, T. B.; Schmitz, R. A., The native microbiome is crucial for offspring generation and fitness of Aurelia aurita. *MBio* **2020,** 11, (6), 10.1128/mbio. 02336-20.
19. Li, J.; Mou, Z.; Yang, H.; Mao, Y.; Yan, Y.; Mo, Z., Isolation and identification the pathogen of Pyropia yezoensis green spot disease. *Progress in FisheryScience* **2018**.
20. Wang, Y., Etiology of skin ulcer syndrome in cultured juveniles of Apostichopus japonicus and analysis of reservoir of the pathogens. *Journal of Fishery Sciences of China/Zhongguo Shuichan Kexue* **2006,** 13, (4).

21. Simidu, U.; Kita-Tsukamoto, K.; Yasumoto, T.; Yotsu, M., Taxonomy of four marine bacterial strains that produce tetrodotoxin. *International journal of systematic and evolutionary microbiology* **1990,** 40, (4), 331-336.
22. Ivanova, E. P.; Romanenko, L. A.; Matte, M. H.; Matte, G. R.; Lysenko, A. M.; Simidu, U.; Kita-Tsukamoto, K.; Sawabe, T.; Vysotskii, M. V.; Frolova, G. M., Retrieval of the species Alteromonas tetraodonis Simidu et al. 1990 as Pseudoalteromonas tetraodonis comb. nov. and emendation of description. *International journal of systematic and evolutionary microbiology* **2001,** 51, (3), 1071-1078.
23. Liu, H.; Zheng, F.; Sun, X.; Hong, X.; Dong, S.; Wang, B.; Tang, X.; Wang, Y., Identification of the pathogens associated with skin ulceration and peristome tumescence in cultured sea cucumbers Apostichopus japonicus (Selenka). *Journal of Invertebrate Pathology* **2010,** 105, (3), 236-242.
24. Topić Popović, N.; Kazazić, S.; Bilić, B.; Babić, S.; Bojanić, K.; Bujak, M.; Tartaro Bujak, I.; Jadan, M.; Strunjak-Perović, I.; Kepec, S., Shewanella spp. from wastewater treatment plant-affected environment: isolation and characterization. *Environmental Science and Pollution Research* **2022,** 29, (55), 82986-83003.
25. Labreuche, Y.; Lambert, C.; Soudant, P.; Boulo, V.; Huvet, A.; Nicolas, J.-L., Cellular and molecular hemocyte responses of the Pacific oyster, Crassostrea gigas, following bacterial infection with Vibrio aestuarianus strain 01/32. *Microbes and Infection* **2006,** 8, (12-13), 2715-2724.
26. Costa, J. C. C. P.; Floriano, B.; Villegas, I. M. B.; Rodríguez-Ruiz, J. P.; Posada-Izquierdo, G. D.; Zurera, G.; Pérez-Rodríguez, F., Study of the microbiological quality, prevalence of foodborne pathogens and product shelf-life of Gilthead sea bream (Sparus aurata) and Sea bass (Dicentrarchus labrax) from aquaculture in estuarine ecosystems of Andalusia (Spain). *Food microbiology* **2020,** 90, 103498.
27. Li, S.-Y.; Huang, Y.-E.; Chen, J.-Y.; Lai, C.-H.; Mao, Y.-C.; Huang, Y.-T.; Liu, P.-Y., Genomics of Ochrobactrum pseudogrignonense (newly named Brucella pseudogrignonensis) reveals a new bla OXA subgroup. *Microbial Genomics* **2021,** 7, (8).
28. Li, S.; Wang, S.; Xie, L.; Liu, Y.; Chen, H.; Feng, J.; Ouyang, L., Identification and optimization of the algicidal activity of a novel marine bacterium against Akashiwo sanguinea. *Frontiers in Marine Science* **2022,** 9, 798544.
29. Wang, L.; Chen, Y.; Huang, H.; Huang, Z.; Chen, H.; Shao, Z., Isolation and identification of Vibrio campbellii as a bacterial pathogen for luminous vibriosis of Litopenaeus vannamei. *Aquaculture Research* **2015,** 46, (2), 395-404.
30. Austin, B.; Austin, D.; Sutherland, R.; Thompson, F.; Swings, J., Pathogenicity of vibrios to rainbow trout (Oncorhynchus mykiss, Walbaum) and Artemia nauplii. *Environmental microbiology* **2005,** 7, (9), 1488-1495.
31. Pérez-Cataluña, A.; Lucena, T.; Tarazona, E.; Arahal, D. R.; Macián, M. C.; Pujalte, M. J., An MLSA approach for the taxonomic update of the Splendidus clade, a lineage containing several fish and shellfish pathogenic Vibrio spp. *Systematic and applied microbiology* **2016,** 39, (6), 361-369.
32. Bruto, M.; James, A.; Petton, B.; Labreuche, Y.; Chenivesse, S.; Alunno-Bruscia, M.; Polz, M. F.; Le Roux, F., Vibrio crassostreae, a benign oyster colonizer turned into a pathogen after plasmid acquisition. *The ISME journal* **2017,** 11, (4), 1043-1052.
33. Song, J.; Liu, X.; Wu, C.; Zhang, Y.; Fan, K.; Zhang, X.; Wei, Y., Isolation, identification and pathogenesis study of Vibrio diabolicus. *Aquaculture* **2021,** 533, 736043.
34. Ramamurthy, T.; Chowdhury, G.; Pazhani, G. P.; Shinoda, S., Vibrio fluvialis: an emerging human pathogen. *Frontiers in microbiology* **2014,** 5, 91.
35. Wang, X.; Zhang, Y.; Qin, G.; Luo, W.; Lin, Q., A novel pathogenic bacteria (Vibrio fortis) causing enteritis in cultured seahorses, Hippocampus erectus Perry, 1810. *Journal of Fish Diseases* **2016,** 39, (6), 765-769.
36. Roux, F. L.; Goubet, A.; Thompson, F.; Faury, N.; Gay, M.; Swings, J.; Saulnier, D., Vibrio gigantis sp. nov., isolated from the haemolymph of cultured oysters (Crassostrea gigas). *International Journal of Systematic and Evolutionary Microbiology* **2005,** 55, (6), 2251-2255.
37. Sicuro, B.; Luzzana, U., The state of Seriola spp. other than yellowtail (S. quinqueradiata) farming in the world. *Reviews in Fisheries Science & Aquaculture* **2016,** 24, (4), 314-325.
38. Harrison, J.; Nelson, K.; Morcrette, H.; Morcrette, C.; Preston, J.; Helmer, L.; Titball, R. W.; Butler, C. S.; Wagley, S., The increased prevalence of Vibrio species and the first reporting of Vibrio jasicida and Vibrio rotiferianus at UK shellfish sites. *Water Research* **2022,** 211, 117942.
39. Huang, B.; Zhang, X.; Wang, C.; Bai, C.; Li, C.; Li, C.; Xin, L., Isolation and characterization of Vibrio kanaloae as a major pathogen associated with mass mortalities of Ark Clam, Scapharca broughtonii, in cold season.

Microorganisms **2021,** 9, (10), 2161.
40. Schaeck, M.; Duchateau, L.; Van Den Broeck, W.; Van Trappen, S.; De Vos, P.; Coulombet, C.; Boon, N.; Haesebrouck, F.; Decostere, A., Vibrio lentus protects gnotobiotic sea bass (Dicentrarchus labrax L.) larvae against challenge with Vibrio harveyi. *Veterinary microbiology* **2016,** 185, 41-48.
41. Farto, R.; Armada, S.; Montes, M.; Guisande, J.; Pérez, M.; Nieto, T., Vibrio lentus associated with diseased wild octopus (Octopus vulgaris). *Journal of Invertebrate Pathology* **2003,** 83, (2), 149-156.
42. Andree, K. B.; Carrasco, N.; Carella, F.; Furones, D.; Prado, P., Vibrio mediterranei, a potential emerging pathogen of marine fauna: investigation of pathogenicity using a bacterial challenge in Pinna nobilis and development of a species-specific PCR. *Journal of applied microbiology* **2021,** 130, (2), 617-631.
43. Torres, M.; Reina, J. C.; Fuentes-Monteverde, J. C.; Fernández, G.; Rodríguez, J.; Jiménez, C.; Llamas, I., AHL-lactonase expression in three marine emerging pathogenic Vibrio spp. reduces virulence and mortality in brine shrimp (Artemia salina) and Manila clam (Venerupis philippinarum). *PloS one* **2018,** 13, (4), e0195176.
44. Mondal, S. K.; Lijon, B.; Reza, R.; Ishika, T., Isolation and identification of Vibrio nereis and Vibrio harveyi in farm raised Penaeus monodon marine shrimp. *Int J Biosci* **2016,** 8, 55-61.
45. Ruiz, P.; Balado, M.; Fuentes-Monteverde, J. C.; Toranzo, A. E.; Rodríguez, J.; Jiménez, C.; Avendaño-Herrera, R.; Lemos, M. L., The fish pathogen Vibrio ordalii under iron deprivation produces the siderophore piscibactin. *Microorganisms* **2019,** 7, (9), 313.
46. Amin, A. R.; Feng, G.; Al-Saari, N.; Meirelles, P. M.; Yamazaki, Y.; Mino, S.; Thompson, F. L.; Sawabe, T.; Sawabe, T., The first temporal and spatial assessment of Vibrio diversity of the surrounding seawater of coral reefs in Ishigaki, Japan. *Frontiers in microbiology* **2016,** 7, 1185.
47. Villamil, L.; Figueras, A.; Aranguren, R.; Novoa, B., Non-specific immune response of turbot, Scophthalmus maximus (L.), experimentally infected with a pathogenic Vibrio pelagius. *Journal of fish diseases* **2003,** 26, (6), 321-329.
48. Guisande, J. A.; Lago, E. P.; Prado, S.; Nieto, T. P.; Seguín, R. F., Genotypic diversity of culturable Vibrio species associated with the culture of oysters and clams in Galicia and screening of their pathogenic potential. *Journal of Shellfish Research* **2008,** 27, (4), 801-809.
49. Verschuere, L.; Heang, H.; Criel, G.; Sorgeloos, P.; Verstraete, W., Selected bacterial strains protect Artemia spp. from the pathogenic effects of Vibrio proteolyticus CW8T2. *Applied and Environmental Microbiology* **2000,** 66, (3), 1139-1146.
50. Ray, A.; Kinch, L. N.; de Souza Santos, M.; Grishin, N. V.; Orth, K.; Salomon, D., Proteomics analysis reveals previously uncharacterized virulence factors in Vibrio proteolyticus. *MBio* **2016,** 7, (4), 10.1128/mbio. 01077-16.
51. Gökbulak, F., Effect of American bison (Bison bison L.) on the recovery and germinability of seeds of range forage species. *Grass and Forage Science* **2002,** 57, (4), 395-400.
52. Qiao, G.; Jang, I.-K.; Won, K. M.; Woo, S. H.; Xu, D.-H.; Park, S. I., Pathogenicity comparison of high-and low-virulence strains of Vibrio scophthalmi in olive flounder Paralichthys olivaceus. *Fisheries science* **2013,** 79, 99-109.
53. Duperthuy, M.; Schmitt, P.; Garzón, E.; Caro, A.; Rosa, R. D.; Le Roux, F.; Lautrédou-Audouy, N.; Got, P.; Romestand, B.; De Lorgeril, J., Use of OmpU porins for attachment and invasion of Crassostrea gigas immune cells by the oyster pathogen Vibrio splendidus. *Proceedings of the National Academy of Sciences* **2011,** 108, (7), 2993-2998.
54. Kesarcodi-Watson, A.; Kaspar, H.; Lategan, M.; Gibson, L., Two pathogens of Greenshell™ mussel larvae, Perna canaliculus: Vibrio splendidus and a V. coralliilyticus/neptunius-like isolate. *Journal of fish diseases* **2009,** 32, (6), 499-507.
55. Robino, E.; Poirier, A. C.; Amraoui, H.; Le Bissonnais, S.; Perret, A.; Lopez-Joven, C.; Auguet, J. C.; Rubio, T. P.; Cazevieille, C.; Rolland, J. L., Resistance of the oyster pathogen Vibrio tasmaniensis LGP32 against grazing by Vannella sp. marine amoeba involves Vsm and CopA virulence factors. *Environmental Microbiology* **2020,** 22, (10), 4183-4197.
56. Ho, H. T.; Lipman, L. J.; Gaastra, W., Arcobacter, what is known and unknown about a potential foodborne zoonotic agent! *Veterinary microbiology* **2006,** 115, (1-3), 1-13.
